# Multilocus Variable-Number Tandem-Repeat Analysis as an Investigation Tool in *Cryptosporidium parvum* Outbreaks in Finland and Sweden in 2022

**DOI:** 10.3390/microorganisms13040821

**Published:** 2025-04-04

**Authors:** Kristiina Suominen, Anni Vainio, Pirkko Hokkanen, Riikka Åberg, Sanna Isosomppi, Eeva Särelä, Wioleta Kitowska, Ana Cristina Gonzalez-Perez, Jukka Ollgren, Ioana Bujila, Karin Troell, Anette Hansen, Mats Lindblad, Ruska Rimhanen-Finne

**Affiliations:** 1 Department of Public Health, Finnish Institute for Health and Welfare, Mannerheimintie 166, 00271 Helsinki, Finland; kristiina.suominen@thl.fi (K.S.); anni.vainio@thl.fi (A.V.); wioleta.kitowska@thl.fi (W.K.); cristina.gonzalezperez@thl.fi (A.C.G.-P.); jukka.ollgren@thl.fi (J.O.); 2 Food Safety Unit, City of Helsinki, Työpajankatu 8, 00580 Helsinki, Finland; pirkko.hokkanen@hel.fi (P.H.); riikka.aberg@hel.fi (R.Å.); 3 Epidemiological Operations Unit, City of Helsinki, Toinen Linja 4 A, 00530 Helsinki, Finland; sanna.isosomppi@hel.fi (S.I.); eeva.sarela@hel.fi (E.S.); 4 ECDC Fellowship Programme, Field Epidemiology Path (EPIET), European Centre for Disease Prevention and Control (ECDC), 169 73 Stockholm, Sweden; 5 ECDC Fellowship Programme, Public Health Microbiology Path (EUPHEM), European Centre for Disease Prevention and Control (ECDC), 169 73 Stockholm, Sweden; 6 Department of Microbiology, Public Health Agency of Sweden, Nobels väg 18, 171 65 Solna, Sweden; ioana.bujila@folkhalsomyndigheten.se; 7 Department of Microbiology, Swedish Veterinary Institute, Ulls väg 2B, 751 89 Uppsala, Sweden; karin.margareta.troell@vetinst.no; 8 Norwegian Veterinary Institute, Elizabeth Stephansens vei 1, 1433 Ås, Norway; 9 Department of Communicable Disease Control and Health Protection, Public Health Agency of Sweden, Nobels väg 18, 171 65 Solna, Sweden; anette.hansen@folkhalsomyndigheten.se; 10 Department of Food Hygiene, Swedish Food Agency, Dag Hammarskjölds väg 56 A, 752 37 Uppsala, Sweden; mats.lindblad@slv.se

**Keywords:** cryptosporidiosis, foodborne, molecular typing, subtype, MLVA scheme, epidemiology, disease outbreaks, cohort study

## Abstract

*Cryptosporidium* is a significant cause of foodborne outbreaks. The 60 kDa glycoprotein gene (*gp60*) is most often used for subtyping *Cryptosporidium* species but is not always sufficient for defining clusters and infections sources. The Multilocus Variable-Number Tandem-Repeat Analysis (MLVA) scheme has been developed to better differentiate between subtypes. A cryptosporidiosis outbreak, with 35 cases, was detected in Finland in September 2022. At the same time, in Sweden, three cryptosporidiosis outbreaks, with 107 cases, were detected, leading to international collaboration. In both countries, salad mixes were suspected as being the outbreak source. In the Finnish outbreak, the suspected salad mixes were produced in Sweden. In the Swedish outbreaks, salad mixes from two different producers were suspected. Twenty-nine patient samples which were positive for *Cryptosporidium parvum* (11 from Finland and 18 from Sweden) were sent for MLVA. The Finnish outbreak samples had different *gp60* subtypes and MLVA profiles compared to the Swedish samples. In our investigation, MLVA differentiated *C. parvum* subtypes in more detail than *gp60* typing. MLVA suggested no connection between the Finnish and Swedish outbreaks. A traceback investigation supported this conclusion. To detect outbreaks and identify infection sources, the timely subtyping of patient samples is crucial and should be implemented in routine surveillance and outbreak investigations.

## 1. Introduction

Protozoa of the genus *Cryptosporidium* are an important cause of gastroenteritis in humans and animals worldwide [1]. The most common species infecting humans are *Cryptosporidium hominis* and zoonotic *Cryptosporidium parvum* [2]. Typical symptoms are watery diarrhea, nausea, vomiting, abdominal pain, and mild fever [3], which develop after an incubation period of one to two weeks [4]. The symptoms of cryptosporidiosis usually self-limit in less than a month; however, relapses occur quite frequently after initial recovery [5]. A serious, even fatal, disease may develop in immunocompromised persons, since no fully effective medication currently exists [4].

Cryptosporidiosis spreads via environmentally robust infectious oocysts, through human-to-human or animal-to-human contact, or via contaminated food or water [6]. *Cryptosporidium* has caused many large waterborne outbreaks worldwide and is considered a significant cause of foodborne outbreaks [7]. *C. parvum* has caused several foodborne outbreaks linked to fresh produce, mainly salads, in both Finland and Sweden [8,9].

Over 40 *Cryptosporidium* species and more than 120 genotypes have been described [10]. Species, genotype, and subtype identification are used to detect infection sources and transmission routes. For the subtyping of *Cryptosporidium* species, the 60 kDa glycoprotein gene (*gp60*) is most often used [11,12]. However, certain subtypes are more commonly found, and subtyping is, therefore, not always sufficient for defining clusters and infection sources [2,13]. A Multilocus Variable-Number Tandem-Repeat Analysis (MLVA) scheme has been shown to have a higher discriminatory power than *gp60* typing, making it potentially useful in investigating outbreaks [14].

On 10 October 2022, the Food Safety Unit of the city of Helsinki, Finland, received information of a suspected foodborne outbreak at a business event at Hotel X on 26–27 September 2022. The participants of the event worked for one company but were from different parts of the country. On 11 October 2022, the Finnish Institute for Health and Welfare (THL) was notified via the national online food and waterborne outbreak register (FWO register). A week later, *Cryptosporidium* sp. was identified as the causative agent of the outbreak. On 24 October, Sweden notified of a *C. parvum* subtype IIaA15G2R1 outbreak via EpiPulse, the European surveillance portal for infectious diseases, leading to collaboration between the Finnish and Swedish authorities. In this report, we describe the Finnish outbreak and the use of the MLVA typing scheme in the outbreak investigation.

## 2. Materials and Methods

### 2.1. Epidemiological Investigation

A retrospective cohort study was conducted to identify the source of the outbreak. A case was defined as a person attending the business event at Hotel X on 26–27 September 2022, participating in at least one of the meals, and developing the symptoms of diarrhea, stomach pain, and/or nausea within 21 days after the event. A link to an online questionnaire was sent to event participants via the company’s contact person on 12 October. The questionnaire contained questions about demographics, the clinical course of the disease, meal attendance, food items consumed during the event, and international travel in the two-week period before the onset of symptoms. Answering the questionnaire was voluntary.

### 2.2. Statistical Analyses

Statistical analyses were conducted using STATA 17.0 software (StataCorp LLC, College Station, TX, USA). Attack rates (AR) and relative risks (RR) with 95% confidence intervals (CI) were calculated for the meals and the foods served during the meals. Fisher’s exact test was used in univariate analysis to determine significance, and a *p*-value < 0.05 was considered statistically significant. For exposures with no unexposed cases or controls, 1 was added to all groups to estimate a rough RR. A notification of the outbreak in Finland was made to EpiPulse on 24 October 2022, in order to identify similar outbreaks in other EU member states.

### 2.3. Environmental and Traceback Investigation

Hotel X, where the business event was held, was newly established and had operated since August 2022. The local environmental health authority conducted an inspection at Hotel X’s restaurant on 11 October 2022. Based on the symptoms and incubation period, *Salmonella* was first suspected as the causative agent. Café Y was identified based on the interview of a case with a matching *C. parvum* genotype with the Swedish outbreak genotype.

Menus and delivery notes were collected, and food samples were taken from the ingredients that were available. The food samples were tested for *Salmonella* and *Escherichia coli*. The local outbreak investigation team conducted a traceback investigation of the suspected food items.

### 2.4. Microbiological Investigation

The patient samples positive for *Cryptosporidium* were sent to THL’s reference laboratory for species determination and *gp60* subtyping. On 20 October 2022, THL released an Infectious Disease Newsletter requesting that the clinical microbiological laboratories send all *Cryptosporidium*-positive patient samples taken after 26 September 2022 to THL in order to assess the magnitude of the outbreak.

The *Cryptosporidium* genus and species (*C. parvum, C. hominis*) were determined using quantitative real-time polymerase chain reaction (qPCR). In addition, 18S rRNA sequencing was used for species identification if the samples remained negative after qPCR and *gp60* assays [15]. For subtyping, *gp60* typing was performed as previously described [15], and the *gp60* PCR product was sent for Sanger sequencing (3730xl DNA Analyzer by Thermo Fisher Scientific, Waltham, MA, USA) to the Institute for Molecular Medicine Finland (Helsinki University, Finland). The subtyping analysis of the sequences was performed at THL by using basic local alignment (BLAST, https://blast.ncbi.nlm.nih.gov/Blast.cgi accessed on 19 February 2025) in the Geneious 2023.2 software (https://www.geneious.com accessed on 12 November 2024).

Based on the *gp60* subtype results, 11 *C. parvum* samples were sent to the *Cryptosporidium* Reference Unit (CRU) at Public Health Wales (Swansea, UK, 9 samples) and the Public Health Agency (PHA, Solna, Sweden, 3 samples) for MLVA typing. One sample was typed both in CRU and PHA. The methods are described in the CRU MLVA assay protocol [16].

### 2.5. Swedish Outbreak Investigation

Three local outbreaks, one company event (event A), one private gathering at home (event B), and one sports event (event C), with altogether 107 cases of *C. parvum* subtype IIaA15G2R1, occurred in three municipalities in Sweden between 25 September and 15 October 2022. Eighteen *C. parvum*-positive patient samples representing all three events were sent to CRU for MLVA typing in order to compare the *C. parvum* subtypes and MLVA profiles to the Finnish findings.

## 3. Results

### 3.1. Clinical Microbiology Findings, Cryptosporidium Species Identification, and gp60 Subtyping

In Finland, bacterial culture and *Cryptosporidium* detection were performed from seven outbreak case samples. The first sample tested for *Cryptosporidium* was taken on 11 October from a person hospitalized due to severe diarrhea. *Cryptosporidium* was found in 4/7 samples. Three samples were sent to THL for typing, and all of them were *C. parvum*. Of them, two were positive for the *C. parvum* subtype IIdA21G1, and one for the *C. parvum* subtype IIaA15G2R1 (Table 1).

In addition to outbreak-related samples, THL received 40 *Cryptosporidium*-positive patient samples from clinical microbiological laboratories between 5 October and 7 November 2022 from different parts of Finland in order to chart if similar *Cryptosporidium* genotypes were found in patients that had not participated in the business event. Of them, four species were identified: *C. parvum*, *Cryptosporidium mortiferum*, *C. hominis*, and *Cryptosporidium ditrichi* (Table 1). For four samples, the species could not be identified, and two samples could not be analyzed due to leakage of the sample and unsuccessful DNA extraction. Of the *C. parvum*-positive samples, five different *gp60* subtypes were identified: IIaA15G2R1, IIaA15G1R1, IIaA13G2R1, IIaA16G1R1, and IIaA19G2R1 (Table 1).

### 3.2. MLVA Typing of Patient Samples

In the Finnish samples, four different MLVA profiles were identified: 4-14-5-7-27-31-15 (4/11), 6-13-2-14-27-31-24 (2/11), 6-13-5-12-18-9-23 (1/11), and in one sample, a mixed result with one locus giving two alleles (4-14-5-7-27-32/35-15) was gained (Table 2). In three samples, the complete MLVA profile could not be determined.

In the Swedish samples, the MLVA profile 6-13-2-14-27-31-24 was identified in 11 samples (Table 2). In three samples, a complete MLVA profile could not be determined, and in four samples, no MLVA profile could be gained. The MLVA profiles in the Finnish and Swedish outbreak samples differed from each other.

### 3.3. Descriptive Epidemiology

**Figure 1:**
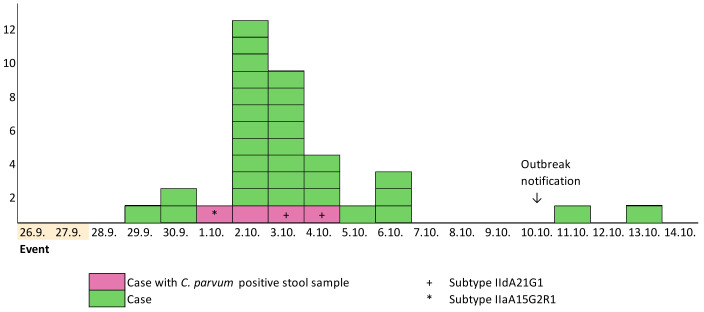
Cryptosporidiosis cases (*N* = 35) by date of symptom onset, September–October 2022, Helsinki, Finland.

Eighty-five guests attended the event and 78% (66/85) responded to the questionnaire. Of them, 53% (35) fulfilled the case definition. For 13 participants, the information about gender and age was missing. Of those who responded, the median age was 44 years (range 26–60 years) and 79% (42/53) were male. The first case developed symptoms on 29 September 2022, and the last on 13 October 2022 (Figure 1).

Seven meals were served during the event, four (breakfast, lunch, afternoon coffee, and dinner) on 26 September, and three (breakfast, lunch, and afternoon coffee) on 27 September. All meals had a separate menu. For food items served during a certain meal, persons not attending that meal or not eating that food item were considered unexposed. All respondents had participated in at least one meal during the event.

The dinner on 26 September was the only meal where all cases had participated; thus, the incubation period was calculated from that date. The median incubation time was seven days (range 3–17 days). The main symptoms reported were diarrhea (97%), abdominal pain (83%), and fatigue (69%). The median duration of symptoms was seven days (range 2–>11 days). At the time of answering the questionnaire, 66% (23/35) still had symptoms. Seven cases had travelled abroad in the two-week period before the onset of symptoms to Germany (6), France (1), and the Netherlands (1). One person had travelled to both Germany and the Netherlands. Travels to Germany were work-related, and the destination was the same, but the dates differed.

After the identification of the MLVA clusters, THL requested information on possible exposures of the cases belonging to clusters from the local authorities. The two non-outbreak-related cases (FI1, FI7) that had a similar MLVA profile to the Swedish outbreak MLVA profile (6-13-2-14-27-31-24) were from different parts of Finland. One of them had eaten a salad mix in Café Y and had not travelled abroad before the illness. Due to the long time period between sampling and the MLVA results, no information was received from the other case.

The four non-outbreak-related cases (FI4, FI5, FI6, FI8) sharing a similar MLVA profile (4-14-5-7-27-31-15) were from three hospital districts. One of the cases had visited a cattle farm where the calves had diarrhea prior to their symptom onset, and had also eaten tartar in a restaurant. One case may have contracted the infection from a symptomatic sibling, who had visited a cattle farm prior to symptom onset. Two cases had not visited cattle farms. Of them, one reported no travels abroad and the other had visited a spa a week prior to symptom onset.

### 3.4. Analytical Epidemiology

Most of the respondents (89%; 59/66) had eaten dinner on 26 September. Of those exposed, 59% (35/59) became ill. The dinner on 26 September was the only meal significantly associated with illness (RR 5.3, 95% CI 0.8–34.11, *p*-value = 0.01) and the only meal in which all cases had participated. An association was found between the cases and eating chicken artichoke salad, green salad, and arugula potatoes at dinner on 26 September; eating bratwurst at breakfast on 27 September; and eating fruit at breakfast on 26 September (Table 3). Of the cases, 97% had eaten green salad, 83% chicken artichoke salad, 74% arugula potatoes, 23% fruits, and 17% bratwurst.

### 3.5. Environmental and Traceback Investigation

The inspection of Hotel X’s premises revealed no irregularities, indicating that all food safety standards were being met. The vegetables and berries used were mainly domestic. The fruits served at the breakfast included watermelon, cantaloupe, and pineapple. Food items served during breakfast were stored separately from other food items. The traceability of foodstuffs, menus, and delivery notes were available. No samples of the foods that were served during the event were stored. The restaurant was reminded of the recommendation that food samples should be taken and stored in a freezer for 2–4 weeks in the case of a foodborne outbreak. Since *Salmonella* was initially the suspected cause, samples of chia seeds and seed mix for salad were taken. The results for *Salmonella* and *E. coli* were negative.

Based on the analytical study, the food items that had the highest association with illness were the chicken artichoke salad, green salad with balsamic vinaigrette, and arugula parmesan potatoes. The chicken artichoke salad contained domestic cooked chicken breast, grilled artichoke, domestic tomato, and imported romaine lettuce. The green salad with balsamic vinaigrette contained salad mix with imported ingredients (radicchio, frisée, and mâche), balsamic, salt, and pepper. The arugula parmesan potatoes contained roasted potatoes and heated arugula oil.

**Figure 2:**
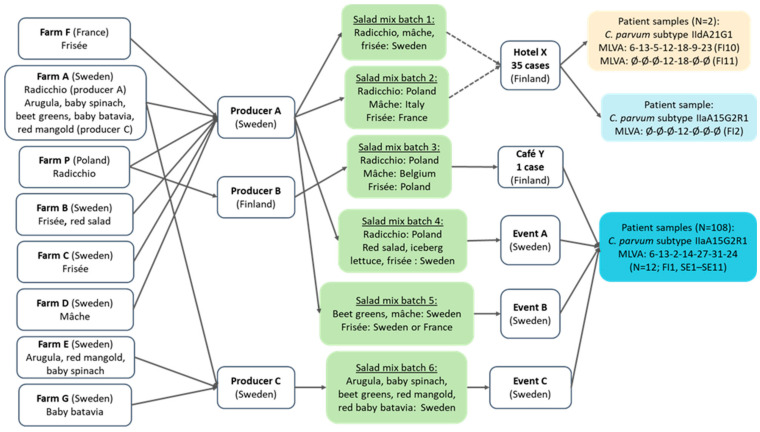
Traceback of salad mix batches linked to *Cryptosporidium parvum* cases at Hotel X and Café Y in Helsinki, Finland, and in three outbreaks in Sweden, from September to October 2022. Either salad mix batch 1 or 2 was served at Hotel X, which is shown with dashed arrows. Abbreviations beginning with FI or SE in parentheses refer to the patient samples in Table 2.

The salad mix served at Hotel X was one of two possible batches produced by producer A (Figure 2). The traceback investigation conducted in Sweden identified various salad mixes (in two outbreaks produced by producer A and in one outbreak by producer C) as potential sources for the outbreaks, but no common ingredient in the salad mixes from the producers A and C could be identified. Frisée salad from Sweden or France was the common ingredient in salad mixes consumed at outbreak events in Finland and Sweden. The radicchios in the salad mix batches 2 (Hotel X) and 3 (Café Y) were traced back to the same farm and batch.

## 4. Discussion

Outbreaks caused by *C. parvum* were identified in September–October 2022 in both Finland and Sweden. Two *gp60* subtypes of *C. parvum* were found in the samples of cases in the Finnish outbreak, one of which was identical to the Swedish outbreak subtype (IIaA15G2R1). In MLVA typing, an incomplete profile of the IIaA15G2R1 sample was gained with one allele identified, but the allele differed from the corresponding allele in the Swedish outbreak samples. MLVA typing of the other *gp60* subtype identified in two of the Finnish outbreak samples (IIdA21G1) yielded one complete and one incomplete profile with two alleles identified. The two alleles identified in the incomplete profile were identical to the corresponding alleles in the complete profile. These findings suggest that the cases in the Finnish outbreak were either not linked to the common event or the source was contaminated by multiple different *C. parvum* types. Similarly, the Finnish and Swedish outbreaks were either not connected or the source was contaminated by multiple different *C. parvum* types.

In four samples of Finnish non-outbreak-related cases, a similar MLVA profile (4-14-5-7-27-31-15) was identified, suggesting a possible common source. Of the cases, two had an epidemiological link to cattle farms, while two did not. In two samples of Finnish non-outbreak-related cases, an identical MLVA profile to the Swedish outbreak profile (6-13-2-14-27-31-24) was gained. Due to the long time between sampling, the MLVA results, and the interviews, exposure information was scarcely available. This highlights the importance of rapid outbreak detection, sampling, and typing of findings, along with conducting patient interviews.

Subtyping patient samples beyond the species level is essential in obtaining microbiological evidence for outbreak investigations. The *gp60* locus in *C. parvum* is highly polymorphic [11], and *gp60* subtyping is often used to differentiate between *C. parvum* isolates [12]. This method can be used in detecting outbreaks, strengthening the epidemiologic connections between cases, and identifying potential sources of cryptosporidiosis [13]. It has been previously used in outbreak investigations in countries such as the UK, New Zealand, Finland, and Sweden [8,9,15,17]. Although *gp60* subtyping is commonly used in outbreak investigations and has strengthened the evidence of the connection to food as the source for the outbreak [18], some *C. parvum* subtypes are very commonly found [2], indicating the need for a subtyping method with high discriminatory power for outbreak investigation. To further discriminate between *C. parvum* subtypes, Robinson et al. [14] developed a multilocus genotyping scheme based on seven variable-number tandem-repeat markers (MLVA). Using MLVA to investigate *C. parvum* cases, Risby et al. [19] identified an outbreak not recognized in routine surveillance and identified more cases belonging to an outbreak already under investigation. MLVA has also been used to strengthen the evidence of an ongoing outbreak investigation [20]. In addition to its high discriminatory power, the advantages of MLVA are its speed and cost-effectiveness when compared to whole-genome sequencing [14].

In the Finnish outbreak, the dinner on 26 September was the only meal in common for all cases. The food items with the highest risk of illness were the chicken artichoke salad, green salad, and arugula potatoes. Of these, the consumption of the green salad explained all but one case, who did not remember whether they ate the green salad or not. A traceback investigation of the suspected vehicle of the Finnish point source outbreak, the green salad mix, showed that one out of two possible salad mix batches had been served to the participants at the event. Both batches were produced in Sweden, one of them with Swedish ingredients and the other with ingredients from various European countries. In Sweden, three local outbreaks in different municipalities with the same MLVA profile occurred in September–October 2022. Salad mixes from two different producers were suspected as the source of these outbreaks. However, no common ingredient in the salad mixes from the two producers could be identified.

Even though salad mixes were not confirmed as the sources of these outbreaks, salads and other fresh produce have previously been implicated in cryptosporidiosis outbreaks [7,8,9]. Fresh produce may be contaminated by fecally contaminated irrigation water, the usage of manure as fertilizer, infected farm workers, or contaminated food contact surfaces [7]. *Cryptosporidium* has many characteristics that contribute to foodborne transmission. For example, the oocysts are resistant to various environmental conditions, do not require maturation before being infectious, are excreted in large numbers during infection, and the infectious dose is low [7]. *Cryptosporidium* oocysts have also been shown to persist and survive on salads during growth [21], as well as during different storage conditions [22]. Also, the washing process has a limited effect in removing the oocysts from the salad leaves, and chlorination does not inactivate them [21].

Our study had limitations. The low number of patient samples obtained in the Finnish outbreak was mainly due to the delayed suspicion of cryptosporidiosis. The first sample for *Cryptosporidium* testing was taken two weeks after the event, when one person was hospitalized due to a severe illness. At that time, many of the cases had already recovered, which probably reduced the willingness to provide samples. This highlights the importance of *Cryptosporidium* testing in outbreak situations and of including the test using gastrointestinal diagnostic panels. MLVA typing was used for the first time in an outbreak investigation in Finland, and the delay in typing led to postponed attempts to obtain exposure data from the non-outbreak-related cases. Furthermore, a complete MLVA profile was not gained for all the patient samples analyzed. Samples of the suspected salad mix batches were not stored, making it impossible to obtain microbiological evidence towards it.

In Finland, cryptosporidiosis is the second most common cause of zoonotic gastroenteritis, along with salmonellosis. Cryptosporidiosis is mostly of domestic origin, and the number of cases has increased in recent years [23]. Still, patient samples are not routinely typed and there is no active case surveillance. *Gp60* subtypes of Finnish patient samples have been recently investigated. In 2019, two *C. parvum* subtypes, IIaA15G2R1 and IIaA13G2R1, were found to be the most common [24]. Similarly, in 2021, *C. parvum* subtype IIaA15G2R1 was the most common subtype identified [15]. Both subtypes, IIaA15G2R1 and IIaA13G2R1, have also been found in calves in Finland [23]. Subtype IIaA15G2R1 is known to be very common in most industrialized countries in humans, as well as in calves and lambs [2,25], leading to difficulties in detecting and solving outbreaks based on *gp60* subtyping alone.

## 5. Conclusions

Our results support the previous findings that MLVA typing has a higher resolution than *gp60* typing and can also differentiate between variants within *C. parvum* subtypes. To detect outbreaks and identify infection sources, the timely subtyping of patient and food samples is crucial and should be implemented in routine surveillance and outbreak investigations.

## Figures and Tables

**Table 1:** *Cryptosporidium* species and *gp60* subtypes of Finnish patient samples (*N* = 37), 5 October–7 November 2022.

*Cryptosporidium* Species (*N*)	*Gp60* Subtype (*n*/*N*)
*C. parvum* (21)	IIaA15G2R1 (10/21) ^1^
	IIdA21G1 (2/21) ^2^
	IIaA13G2R1 (2/21)
	IIaA15G1R1 (1/21)
	IIaA16G1R1 (1/21)
	IIaA19G2R1 (1/21)
	subtype family IIa (1/21)
	NT (3/21) ^3^
*C. mortiferum* (13)	XIVaA20G2T1 (7/13)
	NT (6/13) ^3^
*C. hominis* (2)	IdA16 (1/2)
	IeA11G3T3 (1/2)
*C. ditrichi* (1)	NT ^3^

^1^ Contains one outbreak-related sample. ^2^ Contains two outbreak-related samples. ^3^ NT: non-typeable.

**Table 2:** MLVA profiles of *Cryptosporidium parvum* patient samples from Finland (*N* = 11, 8–27 October 2022) and Sweden (*N* = 14, 25 September–15 October 2022).

Sample	*Gp60* Subtype	MLVA Profile	Epidemiological Link to Producer A’s Salad Mix	Origin of Sample
FI10	IIdA21G1	6-13-5-12-18-9-23	Yes	Finland (outbreak)
FI11	IIdA21G1	Ø-Ø-Ø-12-18-Ø-Ø ^1^	Yes	Finland (outbreak)
FI2	IIaA15G2R1	Ø-Ø-Ø-12-Ø-Ø-Ø ^1^	Yes	Finland (outbreak)
SE1–SE11	IIaA15G2R1	6-13-2-14-27-31-24	Yes	Sweden (outbreak)
SE12	IIaA15G2R1	Ø-13-2-14-27-31-24 ^1^	Yes	Sweden (outbreak)
SE13	IIaA15G2R1	Ø-Ø-2-14-27-31-Ø ^1^	No	Sweden (outbreak)
SE14	IIaA15G2R1	6-Ø-2-14-27-Ø-24 ^1^	Yes	Sweden (outbreak)
FI1	IIaA15G2R1	6-13-2-14-27-31-24	No	Finland
FI7	IIaA15G2R1	6-13-2-14-27-31-24	NA ^2^	Finland
FI4–FI6, FI8	IIaA15G2R1	4-14-5-7-27-31-15	NA ^2^	Finland
FI9	IIaA15G2R1	4-14-5-7-27-32/35-15 (mixed)	NA ^2^	Finland
FI3	subtype family IIa	Ø-Ø-Ø-7-Ø-Ø-Ø ^1^	NA ^2^	Finland

^1^ Ø: non-typeable MLVA loci. ^2^ NA: information not available.

**Table 3:** Food-specific attack rates (AR), relative risks (RR), 95% confidence intervals (CI), and percentage of cases exposed in the *Cryptosporidium parvum* outbreak in Helsinki, Finland, September–October 2022 (*N* = 66). Only exposures with a *p*-value < 0.05 are shown.

Exposure (Meal)	Food Eaten			Food Not Eaten			Relative Risk		*p*-Value	% of Cases (*N* = 35) Exposed
	Total	Cases	AR (%)	Total	Cases	AR (%)	RR	95% CI		
Chicken artichoke salad (Dinner on 26 September)	47	29	62	11	1	9.1	6.8	1.0–45	0.002	83
Green salad ^1^ (Dinner on 26 September)	56	34	61	8	0	0	6.0 ^1^	0.9–39	0.005	97
Arugula potatoes (Dinner on 26 September)	43	26	60	12	2	17	3.6	1.0–13	0.010	74
Bratwurst (Breakfast on 27 September)	6	6	100	52	27	52	1.9	1.5–2.5	0.032	17
Fruit (Breakfast on 26 September)	9	8	89	55	27	49	1.8	1.3–2.6	0.033	23

^1^ Added 1 to all groups in RR calculations.

## Data Availability

The raw/processed data analyzed in this study cannot be shared due to the European General Data Protection Regulation.
